# Developmental Differences in Myocardial Mitochondrial Reticulum Networks in the Offspring Exposed to Diabetic Pregnancy

**DOI:** 10.3390/cells14211698

**Published:** 2025-10-29

**Authors:** Prathapan Ayyappan, Tyler C. T. Gandy, David Sturdevant, Tricia D. Larsen, Pradeeksha Mukuntharaj, Andrew Paulson, Trace A. Christensen, Jeffrey L. Salisbury, Michelle L. Baack

**Affiliations:** 1 Sanford Research, Sioux Falls, SD 57104, USA; gandytyler7@gmail.com (T.C.T.G.); david.sturdevant@sanfordhealth.org (D.S.); tricia.larsen@sanfordhealth.org (T.D.L.); michelle.baack@sanfordhealth.org (M.L.B.); 2 Department of Pediatrics, Sanford School of Medicine, University of South Dakota, Sioux Falls, SD 57105, USA; doctorpradee@gmail.com; 3 Mayo Electron Microscopy Core, Mayo Clinic, Rochester, MN 55905, USA; christensen.trace@mayo.edu (T.A.C.); salisbury@mayo.edu (J.L.S.)

**Keywords:** mitochondrial dynamics, mitochondrial networks, myocardial reticulum, diabetic pregnancy, myocardial development, heart disease

## Abstract

Diabetic pregnancy increases the offspring’s risk of neonatal and adult cardiovascular disease (CVD). We previously used a rat model (Sprague–Dawley) to show that diabetic pregnancy impairs mitochondrial bioenergetics, dynamics, mitophagy, and quality control in the offspring’s heart, and we hypothesized that mitochondrial dysfunction during early development influences the adult myocardium structure to confer cardiometabolic disease risk with aging. Here, we used 3D serial block face-scanning electron microscopy (SBF-SEM) to analyze perinuclear (PN) and intrafibrillar (IF) mitochondrial networks in the left ventricular sections from control and pregestational diabetes-exposed newborn (NB) rats that were three-week-old and four-month-old. Diabetes-exposed myocardium had 50% fewer PN and 20% fewer IF mitochondria at birth but counts increased more rapidly, resulting in no difference at three weeks and 35% more PN and 49% more IF mitochondria by four months. Despite rising counts, mitochondria volumes remained significantly lower at every developmental timepoint. This shows that diabetic pregnancy causes maldevelopment of the myocardial mitochondrial reticulum which likely contributes to adult CVD.

## 1. Introduction

Infants exposed to a diabetic pregnancy are at a higher risk of biphasic cardiovascular disease (CVD) including both structural and functional cardiac disease at birth and premature death from acute myocardial infarction in adulthood [1,2,3]. Evidence from our lab and others shows that in utero exposure to excess circulating fuels of diabetic pregnancy increases the developmental susceptibility to CVD through mitochondria-mediated mechanisms [4,5,6,7]. Using a rat model, we showed that newborns exposed to maternal diabetes had larger hearts, and diastolic and systolic dysfunction [6]. Primary isolated neonatal rat cardiomyocytes (CMs) had impaired respiratory capacity, lower mitochondrial DNA copy number, and fewer, shorter, and poorly dynamic mitochondria by confocal live-cell imaging [5,6,7,8]. This was most pronounced in diabetes-exposed males which also had higher ATP-linked oxygen consumption, more lipid peroxidation, and faster cell death under stress [6,7]. To establish that our model recapitulates the developmental and biphasic nature of cardiac disease that is recognized in humans, newborn offspring exposed to diabetic pregnancy (ODP) and age-matched controls were culled to equal litter size on postnatal day 1 (P1) and cross-fostered for rearing by non-diabetic dams. Cardiac bioenergetics and function were followed into adulthood. Similarly to humans, cardiac enlargement, and diastolic and systolic dysfunction found in newborn ODP were no longer present at weaning (3 weeks) or in young adults (10 weeks or 6 months of age), but cardiac dysfunction reappeared in males by 12 months of age [6]. At a cellular level, ODP had lower mitochondrial DNA (mtDNA) copy number at birth, but as expected, mtDNA increased postnatally when myocardium becomes more reliant on oxidative phosphorylation to support increasing demands [6,8]. As mtDNA copy number increased, cellular bioenergetics improved by 3 weeks with no group differences found at 10 weeks, which is the young adult timepoint. However, by 6 months of age, ODP developed impaired oxidative phosphorylation leading to an exaggerated increase in mtDNA copy number and oxidative damage that preceded a reemergence of cardiac dysfunction in aging rats (12 months) [6]. Bioenergetically, primary CM from 12-month-old ODP had a higher oxygen consumption rate, poor spare respiratory capacity, and faster mitochondria-mediated cell death that could indicate a greater risk of myocardial damage following metabolic stress [6].

Given these previous findings, we hypothesized that mitochondrial dysfunction, including impaired dynamics during cardiac development, would result in maldevelopment of the mitochondrial reticulum (the interconnected network of mitochondria), contributing to an age-related susceptibility to myocardial disease in ODP. The objective of this study was to use 3D serial block face-scanning electron microscopy (SBF-SEM) to evaluate the postnatal development of the mitochondrial reticulum and determine whether pregestational diabetes incites developmental aberrations that could explain age-related risks in heart disease susceptibility.

## 2. Methods

### 2.1. Animal Care

**Figure 1:**
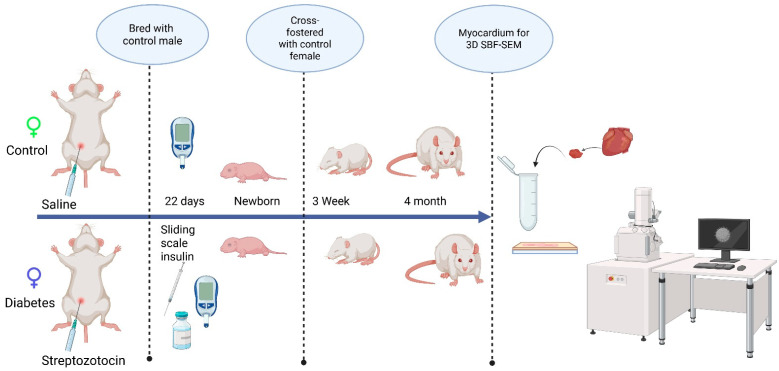
The schematic representation of the experimental design, examining the developmental differences in the mitochondrial reticulum network in the heart.

This study followed guidelines set forth by the Animal Welfare Act and the National Institutes of Health Guide for the Care and Use of Laboratory Animals and was under approval from the Sanford Research Institutional Animal Care and Use Committee (Protocol #170-06-23B and 153-10-21B). All animals (Sprague–Dawley rats) were housed in a temperature-controlled, light–dark-cycled facility with free access to water and chow (TD2018 Teklad, Envigo; 18% fat, 24% protein, 58% carbohydrates) throughout the experiment. Prior to breeding, female rats (10 weeks of age) received an intraperitoneal injection of 65 mg/kg streptozotocin (Sigma-Aldrich, Inc., St. Louis, MO, USA) to induce pregestational diabetes and the control rats received citrate-buffered saline (20 mM, Thomas Scientific, Swedesboro, NJ, USA). Female rats that did not manifest diabetes, defined as a blood glucose < 200 mg/dL within 48 h after streptozotocin injection, were excluded from the study. After confirming the induction of diabetes, female rats were bred with healthy male rats and monitored by daily vaginal swab for spermatozoa. When spermatozoa were first present, timed pregnancy started as embryonic day 0 (E0). With a goal to keep blood glucose levels at 200–400 mg/dL, dams were partially treated with sliding scale insulin (regular and glargine, Eli Lilly and Co., Indianapolis, IN, USA) two times per day. Whole blood sampling from a tail nick was performed to measure glucose at least twice daily and ketones (βHOB) daily (Precision Xtra glucometer and ketone meter, Abbott Laboratories, Abbott Park, IL, USA). Dams were allowed to deliver spontaneously to yield offspring of both sexes from two distinct groups: the control group and the diabetes-exposed group. After equalizing the litter size, the offspring were cross-fostered on P1 to normal-timed pregnant dams fed a control diet that had delivered 0–7 days prior to the corresponding experimental offspring. To ensure that exposure to diabetes was only prenatal, all pups were weaned to a control diet before being aged to the preset timepoints (3 weeks and 4 months). On P1, the 3-week, and 4-month timepoints, male offspring hearts were collected under 5% isoflurane anesthesia and stored at −80 °C until analysis. The scheme of experimental procedures is presented in Figure 1.

### 2.2. Serial Block Face-Scanning Electron Microscopy (SBF-SEM)

Myocardium from control and diabetes-exposed rats was evaluated at P1 (newborn), 3 week (weaning), and 4 month (adult) developmental timepoints using 1 mm^3^ sections of the left ventricular septum fixed and prepared for SBF-SEM as previously detailed [9]. Specifically, myocardial samples were fixed in 0.15 M cacodylate buffer containing 2 mM of CaCl_2_, 2% of glutaraldehyde and 2% of paraformaldyhyde for 24 h. Fixed samples were washed in 0.15 M of cacodylate buffer and incubated at room temperature in 2% of osmium tetroxide in 0.15 M of cacodylate buffer for 1.5 h, followed by another incubation in cacodylate buffer (0.15 M) containing 2.5% of potassium ferracyanide and 2% of osmium tetroxide for another 1.5 h at room temperature. Samples were rinsed with water and incubated in 1% of thiocarbohydrazide for 45 min at 50 °C. After another round of rinsing with water, samples were incubated sequentially in 2% of osmium tetroxide in water for 1.5 h at room temperature, 1% of aqueous uranyl acetate overnight at 4 °C, and in 7% of lead aspartate solution for 1 h at 50 °C with several rinses in water between each reagent. Following dehydration through a series of ethanol and acetone washes, samples were infiltrated and eventually embedded in polyepoxide resin, and Durcapan (EMS, Hatfield, PA, USA), and polymerized in a 60 °C oven for a minimum of 24 h. To prepare embedded samples for placement into the SEM and subsequent imaging, 1 mm^3^ pieces were roughly trimmed and mounted to 8 mm aluminum stubs using epoxy EPO-Tek (EMS, Hatfield, PA, USA). Then the mounted sample was again trimmed to a 0.5 mm × 0.5 mm × 1 mm tall tower. To assist in charge dissipation, the entire stub along with the trimmed sample was coated with gold palladium. Then the coated samples were inserted into a VolumeScope for SBF-SEM (Thermo Fisher, Waltham, MA, USA) at the Mayo Electron Microscopy Core and allowed to acclimate to high vacuum for 12 h prior to the start of the imaging. Approximately 300 high resolution images/section at 50 nm increments were captured by SBF-SEM in a low-vacuum environment using a beam energy of 3.0 kV with a current of 100 pA and a scanning dwell time of 2 µs and 10 nm pixel size.

**Figure 2:**
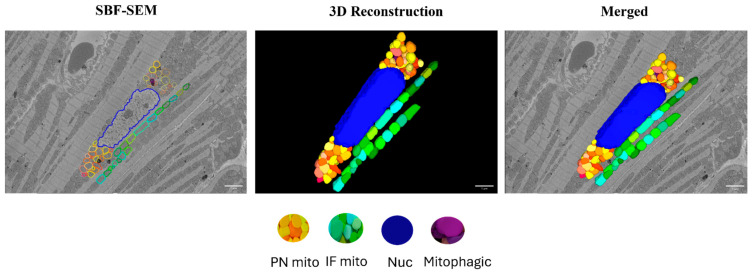
Three-dimensional reconstruction of mitochondrial reticulum in the myocardium from a 4-month-old male rat. Nucleus (blue), PN mitochondria (warm colors), and IF mitochondria (cool colors) were traced in the SBF-SEM images obtained from the left ventricular septum of the myocardium. Purple color indicates mitochondria undergoing mitophagy. Scale bars: 1 µm.

Images were aligned using the Amira (2020.2) software and 3D reconstructions were created using Image J (20130715) and the Autodesk Inventor Professional 2022/2023 software. Interfibrillar (IF) mitochondria that reside directly between two myofibril units were counted within a 10 × 10 µm grid over a stack of 10 images per biological replicate and analyzed separately using the Image J software. Perinuclear (PN) mitochondria which are groups of mitochondria that are directly adjacent to the nucleus and each other on both sides of the nucleus were counted over a stack of 10 images per biological replicate and averaged. For calculating mitochondrial volume, volume from 10 PN and 10 IF mitochondria per myocardial section were measured using the Reconstruct software (1.1.0.0). All the quantifications were performed from two regions of interest (ROIs) per biological replicate. All the image analysis was performed in blinded fashion. Tracing of the perinuclear (PN) and interfibrillar (IF) mitochondria and the subsequent 3D reconstruction of the SBF-SEM images of the myocardium are represented in Figure 2.

### 2.3. Statistical Analysis

Group comparisons of mitochondria count and volume at each timepoint were performed using Student’s *t*-test in PRISM 9 (Graphpad Software) and the data are represented as mean ± SEM (*n* = 3 animals/group). Local regression fitting by locally estimated scatterplot smoothing (LOESS) was used to examine the difference in mitochondrial count and volume between the groups over time with 95% CI and alpha of 0.05.

## 3. Results

### 3.1. Myocardial Mitochondria Count in Control and Diabetes-Exposed Offspring over Time

**Figure 3:**
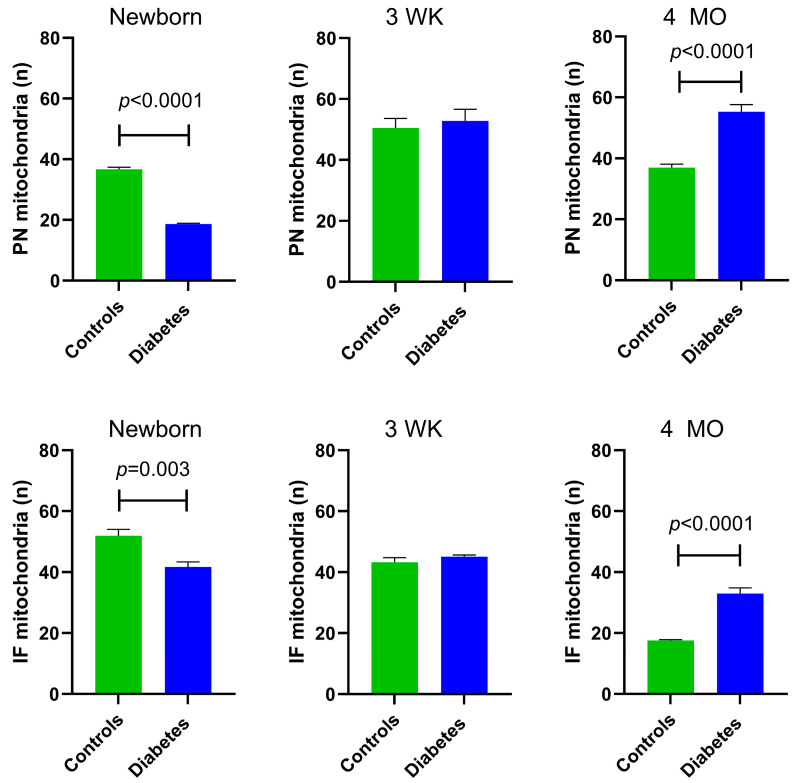
Myocardial mitochondrial counts in control and diabetes-exposed male offspring. The number of perinuclear (PN) mitochondria per cluster were counted and averaged over 10 slices for two ROIs per myocardial section. The number of interfibrillar (IF) mitochondria within a standardized 10 × 10 µm grid were counted and averaged in similar manner. Bar graphs represent mean ± SEM; *n* = 3 animals/group. *p* values denote group difference at each timepoint by Student’s *t*-test.

At P1, diabetes-exposed newborn myocardium had 50% fewer PN (*p* < 0.0001) and 20% fewer IF mitochondria (*p* = 0.003). By 3 weeks of age, there were no longer exposure-related differences in PN or IF mitochondria count. Interestingly, at the 4-month adult timepoint, diabetes-exposed offspring had a 35% higher number of PN mitochondria (*p* < 0.0001) and 49% more IF mitochondria (*p* < 0.0001) compared to control offspring (Figure 3).

### 3.2. Mitochondria Volume in Control and Diabetes-Exposed Offspring Hearts over Time

**Figure 4:**
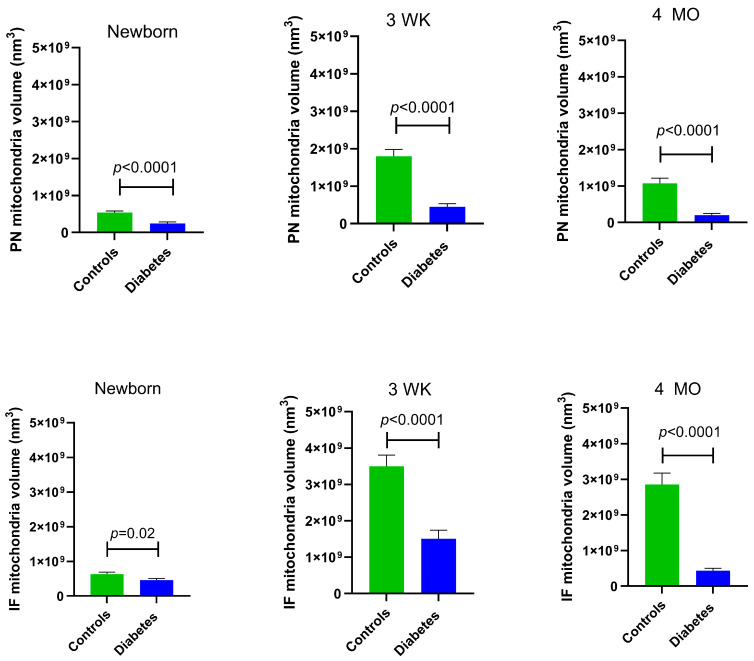
Myocardial mitochondrial volume was significantly different in diabetes-exposed male offspring at different developmental time periods. Average volume of mitochondria from 10 slices for two ROIs per myocardial section was used for group comparisons. Bar graphs represent mean ± SEM; *n* = 3 animals/group. *p* values denote group differences at each timepoint by Student’s *t*-test.

At all developmental timepoints, PN and IF mitochondria volume were significantly lower in diabetes-exposed myocardium compared to the control myocardium (*p* = 0.02 for newborn IF mitochondria and *p* < 0.0001 for all others). Of importance, despite having a higher number of mitochondria, 4-month-old adult diabetes-exposed offspring have a significantly lower mitochondria volume compared to control offspring (Figure 4).

### 3.3. Number and Volume Vary by Mitochondria Type Across Development in Both Control and Diabetes-Exposed Offspring Myocardium

**Figure 5:**
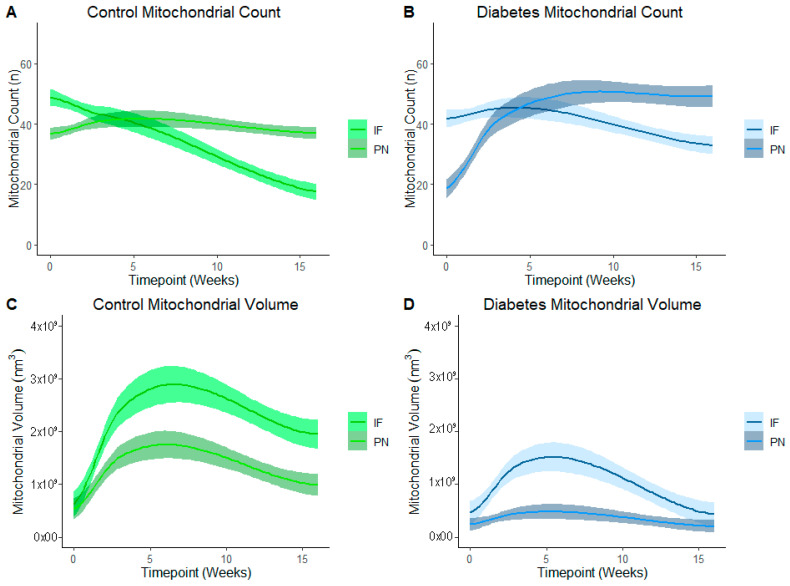
Developmental differences in mitochondrial reticulum in terms of number and volume of mitochondria in control and in diabetes-exposed myocardium in male offspring over time. Local regression fitting by locally estimated scatterplot smoothing (LOESS) was used to compare a moving average over developmental timepoints. Each line represents a regression for either IF or PN, and shaded bands represent a 95% confident interval that the true regression line falls within. Wherever the shaded regions are not touching, there is a significant difference between the bands (*p* = 0.05) at the point on the *x*-axis.

Figure 5 represents PN and IF mitochondrial counts and volume in control (Figure 5A,C) and diabetes-exposed (Figure 5B,D) offspring myocardium across predetermined developmental timepoints. LOESS was used to compare a moving average over developmental timepoints. Each line represents a regression for either PN or for IF mitochondria, and shaded bands represent a 95% confident interval that the true regression line falls within. Wherever the shaded regions are not touching, there is a significant difference between the bands (*p* = 0.05) at the specified point on the *x*-axis. At birth, normal (control) myocardium had more IF than PN mitochondria, but the number of IF mitochondria fell as their volume increased over time. Thereafter, IF mitochondria volume was significantly higher than PN volume. This was expected because IF mitochondria normally fuse to form interconnected networks between myofibrils to make up the highly organized mitochondria reticulum, which is a power grid-like arrangement that promotes efficient energy production. Given their varied role in nuclear communication, PN mitochondria counts were lower than IF counts at birth, but both number and volume of PN mitochondria increased after birth to a peak at 3 weeks of age. Thereafter, normal myocardium has higher PN mitochondria counts, but volume remains significantly lower than the network of IF mitochondria.

In diabetes-exposed offspring, IF and PN mitochondria patterns follow the same trajectory. However, compared to controls, IF counts do not decline at the same rate and there is a severely blunted peak IF volume at 3 weeks which suggests an impairment in fusion and networking. This results in a similar volume of PN and IF mitochondria at the 4-month timepoint.

### 3.4. Effects of Diabetes-Exposure on Development of the Myocardial Mitochondrial Reticulum

**Figure 6:**
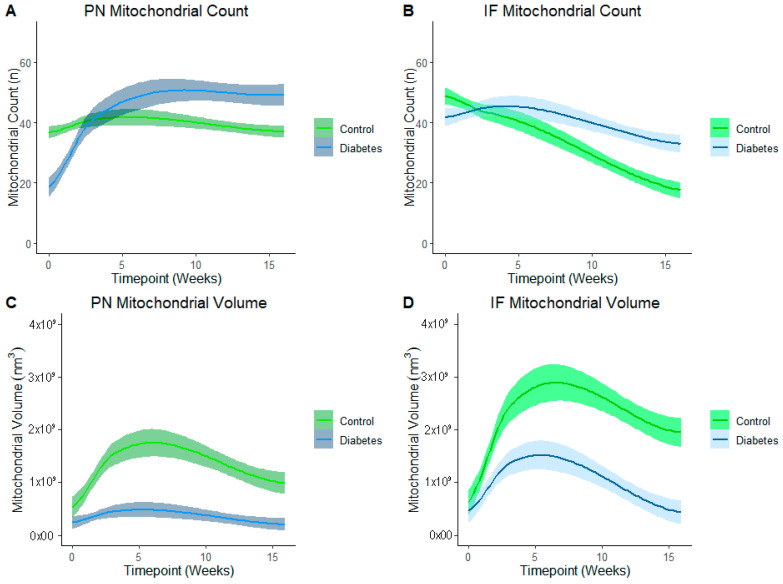
Diabetes-exposure altered the development of myocardial mitochondrial reticulum compared to controls. LOESS analysis shows that both the PN and IF mitochondrial volume was significantly different in diabetes-exposed myocardium in male offspring at different developmental stages regardless of the mitochondrial counts. Each line represents a regression for either the control or diabetes-exposed group, and shaded bands represent a 95% confident interval that the true regression line falls within. Wherever the shaded regions are not touching, there is a significant difference between the bands (*p* = 0.05) at the point on the *x*-axis.

**Figure 7:**
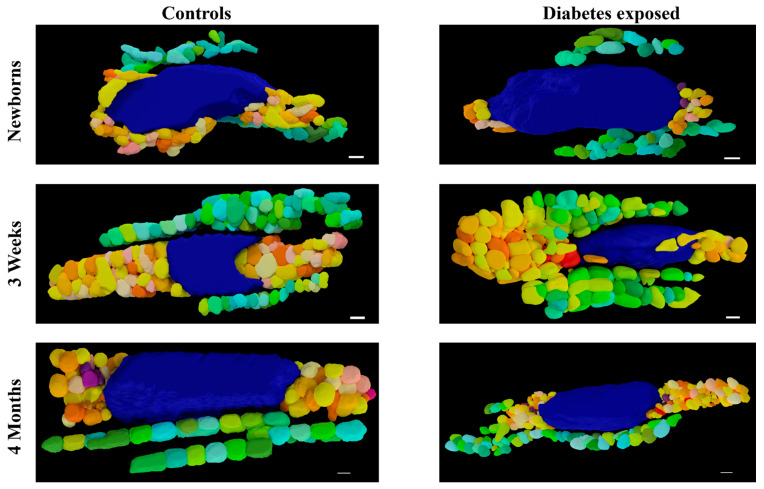
Three-dimensional reconstruction of SBF-SEM images of control and diabetes-exposed myocardium in male offspring at different developmental stages. Detailed 3D models of control and diabetes-exposed myocardium reveal that despite having higher number of IF mitochondria, their volume was significantly reduced over time. Blue color represents nucleus, warm colors represent PN mitochondria, and cool colors represent IF mitochondria. Purple color represents electron dense material that indicates mitophagy. Scale bars: 1 µm.

LOESS analysis by the exposure group shows that development of the mitochondrial reticulum was drastically affected by diabetes-exposure with the number and volume of both PN and IF mitochondria being significantly different across nearly every developmental timepoint (Figure 6). Specifically, both PN and IF mitochondria counts were lower in diabetes-exposed myocardium at birth but counts increased more robustly than controls, resulting in no significant difference at 3 weeks of age and significantly higher counts in adulthood. Regardless of the count, mitochondrial volume was significantly lower in diabetes-exposed myocardium at every timepoint. A high number of small mitochondria suggests an imbalance of the mitochondria fusion and fission that disrupts networking. Qualitatively, 3D reconstruction of SBF-SEM images (Figure 7) revealed obvious developmental defects in the mitochondrial reticulum network in diabetes-exposed myocardium compared to controls (Videos S1–S6 in Supplementary Files).

## 4. Discussion

Mitochondrial dynamics or the ability for mitochondria to undergo fusion and fission enables real-time mitochondrial modifications in ultrastructure or network morphology, replication, culling, complex assembly, and mtDNA turnover that ensures mitochondrial quality control and metabolic flexibility to meet changing energetic demands of the cell [10]. Fusion and fission also regulate non-dynamic cell processes including mitochondrial ER/SR communication, calcium flux, mitophagy, and mitochondria-mediated cell death [11,12]. Given the high energy demands of the heart, mitochondrial dynamics is a well-recognized key modulator of both cardiac development and disease [13]. Our past work was instrumental in demonstrating that maternal diabetes perturbs mitochondrial dynamics, impairs cardiomyocyte (CM) bioenergetics, and decreases cardiac contractility in newborn ODP; and maternal high-fat diet exacerbates the newborn phenotype, causing myocardial lipid accumulation, oxidative damage, and higher perinatal mortality [5,8]. Developing CMs have very dynamic mitochondria with well-balanced fusion and fission. Excess fission or impaired fusion results in a pro-fission phenotype with fragmented, poorly charged, and dysfunctional mitochondria that produce ROS and signal mitophagy. When in excess, this induces cell death. Using a rat model, we previously showed that diabetes-exposed neonatal CM had 50% fewer fusion and 30% fewer fission events, resulting in a pro-fission ratio of ~1.8 which was significantly higher in male, but not female offspring [5]. Mechanisms of impaired dynamism were protein-regulated and sex-specific, with males having more post-translational modifications that activate fission proteins and females having a higher expression of fusion proteins which may confer relative cardioprotective effects [5].

Using a similar model, we followed offspring into adulthood and showed that like in humans, cardiac function in diabetes-exposed rats improved after birth, leaving no apparent differences in very early adulthood, but as myocardial metabolism becomes increasingly reliant on oxidative phosphorylation during maturation, diabetes-exposed CM had impaired ability to ramp up respiration which preceded a decline in cardiac function in aging males [6]. Furthermore, male, but not female ODP had faster mitochondrial membrane potential loss and cell death with metabolic stress which translates to greater myocardial damage following ischemia [6,7]. This earlier work established that impaired mitochondrial dynamics is a fuel-regulated and sex-specific consequence of diabetic pregnancy that is associated with adult CVD in male ODP. We hypothesized that a higher fission/fusion ratio found in newborn males could alter the trajectory of normal myocardial mitochondrial network development and set the stage for the pro-apoptotic cellular phenotype which influences the risk of CVD in adulthood. The heart’s putative physically connected mitochondrial network enables rapid communication and distribution of potential energy throughout the cell [14,15]. This is necessary to support efficient energy production to maintain continuous contractility. Indeed, myocardium is unique with multiple subpopulations of mitochondria such as perinuclear (PN), interfibrillar (IF), and subsarcolemmal (SS) mitochondria, which has different properties [16]. PN mitochondria form spherical dense clusters surrounding the nucleus whereas IF mitochondria are in the longitudinal rows between myofibrils; SS mitochondria are seen under sarcolemma [16,17]. PN mitochondria are mobile and found to participate in mitochondrial biogenesis, dynamics, and turnover. IF mitochondria, the major population, is relatively static and restricted in their networked positions. They have higher substrate oxidation capacity along with increased activities of major enzymes responsible for oxidative phosphorylation and provide bioenergetic support for cardiac contraction and mitochondrial interactions with the cytoskeleton and sarcoplasmic reticulum [18,19,20]. While IF mitochondria are responsible for the generation of ATP for myocardial contraction; SS mitochondria support the transport of electrolytes and metabolites via sarcolemma [20]. Since the adult heart is characterized by well-developed mitochondrial reticulum networks that support high energy demand in the heart, it is important to understand the developmental and/or functional consequences of the prenatal exposure of maternal diabetes on the formation of these myocardial mitochondrial networks in the offspring and whether the network improves or remains disrupted postnatally over aging.

This study shows that the number of PN and IF mitochondria in the heart were significantly lower in the diabetes-exposed newborns compared to control newborns. PN mitochondria appears to play a significant role in the regulation of the mitochondrial network to meet the metabolic needs of the cell whereas IF mitochondria support contractile function by generating ATP [16,17]. Since PN mitochondria are involved in biogenesis and fission/fusion dynamics, this result is not surprising as it also correlates with our previous study results that show that mitochondrial dynamics and bioenergetics were altered in neonatal cardiomyocytes exposed to maternal diabetes and a high-fat diet [5,8]. A lower number of IF mitochondria in diabetes-exposed offspring could also account for bioenergetic dysfunction and compromised myocardial function as we observed from our previous studies [6,7]. However, in mid-to-late adulthood, diabetes-exposed offspring develop impaired myocardial energetics and a compensatory exaggerated increase in mitochondrial counts over time. In the current study, the number of myocardial PN and IF mitochondria were significantly higher at 4 months of age in diabetes-exposed offspring compared to control offspring. The relative abundance of PN and IF mitochondria in the offspring exposed to diabetic pregnancy at 4 months of age (adulthood) points towards impaired mitochondrial dynamics and networking over time, rather than impaired biogenesis. A higher number of small, fragmented, and poorly connected mitochondria can lead to more reactive oxygen species (ROS) formation, mitophagy, and cardiomyocyte death, as seen in aged male ODP from our model [6].

As proposed, we found a reduction in the volume of PN and IF mitochondria in the diabetes-exposed offspring at all the developmental timepoints. Interestingly, even with a relative abundance of IF and PN mitochondria in the diabetes-exposed offspring at 4 months of age, the overall volume was significantly reduced. Mitochondria increase their number via biogenesis and mitochondrial fission, which is also crucial for mitophagy. On the other hand, mitochondrial fusion is crucial for increasing the mitochondrial volume and fusion-mediated mitochondrial connectivity is important to facilitate cardiac contractile function across the lifespan. An imbalance between mitochondrial fission and fusion could result in more fragmented mitochondria, eventually leading to increased ROS generation and mitophagy [21]. Our study results clearly indicate that there is a disruption in the mitochondrial network leading to poor connectivity in the myocardium that could explain cardiac dysfunction and faster cardiomyocyte death in the male offspring exposed to diabetic pregnancy.

LOESS analysis in our study established the developmental differences in the PN and IF mitochondrial count and their volume over time in both control and diabetes-exposed offspring that could account for impaired mitochondrial reticulum development. Others previously reported that myocardial mitochondria undergo continuous changes in their morphology and networks during development, which is closely related to mitochondrial function [22]. Higher number of PN mitochondria along with less volume and relatively higher number of IF mitochondria with smaller size during adulthood in diabetes-exposed offspring suggests that alterations in mitochondrial dynamics lead to lasting effects across aging that could impair bioenergetics and contractile function. Others have reported that cardiac mitochondrial size was reduced in multiple pathological conditions, including diabetic cardiomyopathy which is associated with heart failure [17,23]. Interestingly, humans with diabetes also have fragmented cardiac mitochondrial networks due to impaired mitochondrial fusion [24]. Taken together, our results show a link between impaired mitochondrial dynamics and mitochondrial networking that could explain the mechanisms of developmentally programmed adult heart disease in male ODP.

### Limitations and Future Directions

In the present study, we only evaluated the developmental differences in mitochondrial reticulum over time in male hearts. This is because sex-specific differences in mitochondrial function, mitochondrial dynamics, and cardiomyocyte death were identified in males, not in females, during our previous studies [6,7]. Further studies are warranted to see the sex-specific differences in mitochondrial reticulum development at different developmental timepoints by including more biological replicates from both males and females. Additional work is required to assess the downstream effects of fragmented mitochondrial networks on calcium-handling proteins like SERCA 2A as it relates to diastolic and systolic dysfunction [25,26]. There is also a need to test potential mitigating therapies such as SGLT2 inhibitors, small molecule activators of mitochondrial fusion, and mitochondrial antioxidants like Coenzyme Q10 or mitoquinone which ameliorate mitochondria-mediated cardiomyopathy in other conditions [27,28,29,30,31,32].

## 5. Conclusions

In summary, this study establishes that fetal exposure to pregestational diabetic pregnancy causes maldevelopment of the mitochondrial reticulum that persists into adulthood. Male ODP have fewer and smaller mitochondria at birth, followed by an exaggerated increase in mitochondrial counts, but a lower mitochondrial volume with poor connectivity. The increase in mitochondria number between birth and 3 weeks could explain why newborns exposed to diabetic pregnancy have cardiac dysfunction that improves after birth, but over time cardiac function declines and males have a higher risk of myocardial infarction with aging. These findings along with our previous confocal, mtDNA copy number, bioenergetics, and echocardiographic findings over time strongly suggest that diabetes-mediated alterations in mitochondria play a critical role in developmentally programmed adult cardiac disease.

## Data Availability

Any data not included in the manuscript or Supplementary Files are available from the corresponding author upon request.

## Supplementary Materials

The following supporting information can be downloaded at: https://www.mdpi.com/article/10.3390/cells14211698/s1, Video S1: Three dimensional reconstructed myocardial mitochondrial reticulum using SBF_SEM images from the Control newborns (NB_CDCB) rats; Video S2: Three dimensional reconstructed myocardial mitochondrial reticulum using SBF_SEM images obtained from the newborn rats exposed to maternal diabetes (NB_DM); Video S3: Three dimensional reconstructed myocardial mitochondrial reticulum using SBF_SEM images obtained from the control 3 week old rats (3 Wk_CDCB); Video S4: Three dimensional reconstructed myocardial mitochondrial reticulum using SBF_SEM images obtained from the control 3 week old rats exposed to maternal diabetes (3 Wk_DM); Video S5: Three dimensional reconstructed myocardial mitochondrial reticulum using SBF_SEM images obtained from the control 4 month old control rats (4 Months_CDCB); Video S6: Three dimensional reconstructed myocardial mitochondrial reticulum using SBF_SEM images obtained from the 4 month old rats exposed to maternal diabetes (4 Months_DM).
