# Supplementary material for: Developmental Differences in Myocardial Mitochondrial Reticulum Networks in the Offspring Exposed to Diabetic Pregnancy
Source: Cells. 2025 Oct 29;14(21):1698. doi: 10.3390/cells14211698 (PMC12609361; doi:10.3390/cells14211698)
Supplement: Supplementary file 1 [file cells-14-01698-s001.zip › Legends to videos.pdf]

Supplementary File

Legends to videos

Video S1: 3D-reconstructed myocardial mitochondrial reticulum using SBF_SEM images from the Control newborns (NB_CDCB) rats

Video S2: 3D-reconstructed myocardial mitochondrial reticulum using SBF_SEM images obtained from the newborn rats exposed to maternal diabetes (NB_DM).

Video S3: 3D-reconstructed myocardial mitochondrial reticulum using SBF_SEM images obtained from the control 3 week old rats (3 Wk_CDCB).

Video S4: 3D-reconstructed myocardial mitochondrial reticulum using SBF_SEM images obtained from the control 3 week old rats exposed to maternal diabetes (3 Wk_DM).

Video S5: 3D-reconstructed myocardial mitochondrial reticulum using SBF_SEM images obtained from the control 4 month old control rats (4 Months_CDCB).

Video S6: 3D-reconstructed myocardial mitochondrial reticulum using SBF_SEM images obtained from the 4 month old rats exposed to maternal diabetes (4 Months_DM).
